# Children with Spastic Cerebral Palsy Experience Difficulties Adjusting Their Gait Pattern to Weight Added to the Waist, While Typically Developing Children Do Not

**DOI:** 10.3389/fnhum.2016.00657

**Published:** 2016-12-23

**Authors:** Pieter Meyns, Leen Van Gestel, Lynn Bar-On, Marije Goudriaan, Hans Wambacq, Erwin Aertbeliën, Herman Bruyninckx, Guy Molenaers, Paul De Cock, Els Ortibus, Kaat Desloovere

**Affiliations:** ^1^Department of Rehabilitation Medicine, MOVE Research Institute Amsterdam, VU University Medical CenterAmsterdam, Netherlands; ^2^Department of Rehabilitation Sciences, Faculty of Kinesiology and Rehabilitation SciencesKU Leuven, Leuven, Belgium; ^3^Clinical Motion Analysis Laboratory, University Hospital LeuvenLeuven, Belgium; ^4^Division of Production Engineering, Machine Design and Automation, Faculty of Engineering, Department of Mechanical Engineering, KU LeuvenLeuven, Belgium; ^5^Department of Pediatric Orthopaedics, University Hospital LeuvenLeuven, Belgium; ^6^Faculty of Medicine, Department of Musculoskeletal SciencesKU Leuven, Leuven, Belgium; ^7^Centre for Developmental Disabilities, University Hospital LeuvenLeuven, Belgium

**Keywords:** Cerebral Palsy, gait, weight, body mass, EMG, muscle weakness

## Abstract

The prevalence of childhood overweight and obesity is increasing in the last decades, also in children with Cerebral Palsy (CP). Even though it has been established that an increase in weight can have important negative effects on gait in healthy adults and children, it has not been investigated what the effect is of an increase in body weight on the characteristics of gait in children with CP. In CP, pre and post three-dimensional gait analyses are performed to assess the effectiveness of an intervention. As a considerable amount of time can elapse between these measurements, and the effect of an alteration in the body weight is not taken into consideration, this effect of increased body weight is of specific importance. Thirty children with the predominantly spastic type of CP and 15 typically developing (TD) children were enrolled (age 3–15 years). All children underwent three-dimensional gait analysis with weight-free (baseline) and weighted (10% of the body weight added around their waist) trials. Numerous gait parameters showed a different response to the added weight for TD and CP children. TD children increased walking velocity, step- and stride length, and decreased double support duration with a slightly earlier timing of foot-off, while the opposite was found in CP. Similarly, increased ranges of motion at the pelvis (coronal plane) and hip (all planes), higher joint angular velocities at the hip and ankle, as well as increased moments and powers at the hip, knee and ankle were observed for TD children, while CP children did not change or even showed decreases in the respective measures in response to walking with added weight. Further, while TD children increased their gastrocnemius EMG amplitude during weighted walking, CP children slightly decreased their gastrocnemius EMG amplitude. As such, an increase in weight has a significant effect on the gait pattern in CP children. Clinical gait analysts should therefore take into account the negative effects of increased weight during pre–post measurements to avoid misinterpretation of treatment results. Overweight and obesity in CP should be counteracted or prevented as the increased weight has detrimental effects on the gait pattern.

## Introduction

According to the World Health Organization (WHO), in 2014 more than 1.9 billion adults were overweight. Of these over 600 million were obese (World Health Organization, 2016b). Furthermore, the worldwide prevalence of obesity has more than doubled between 1980 and 2013 (Finucane et al., 2011; Ng et al., 2014).

Obesity has an important impact on reduced quality of life (Taylor et al., 2013) and public health (Visscher and Seidell, 2001), as it is related to the development of several non-communicable diseases such as cardiovascular disease, type 2 diabetes mellitus, and osteoarthritis (World Health Organization, 2016b).

Similarly, in children an increase in overweight and obesity has been observed over the last decades (de Onis et al., 2010). This is not only the case in typically developing children (TDc), but also in children that already present with a pathology; e.g., children with Cerebral Palsy (CPc) (Rogozinski et al., 2007; Park et al., 2011).

Cerebral Palsy is the most common developmental cause of physical disability in the world (Aisen et al., 2011), with a prevalence of 2–3 in 1000 live births (Odding et al., 2006). It originates from non-progressive impairments to the brain before, during or shortly after birth (Bax et al., 2005), resulting in persistent (primary) motor and sensory impairments such as abnormal motor control, muscle strength, and/or muscle tone. As a result of abnormal muscle activity or loading of the bones, secondary impairments can develop over time, such as shortened muscles which will restrict the joint range of motion. These primary and secondary impairments result in a pathological gait pattern in CPc.

Many treatment modalities in CP are aimed at improving the gait pattern, by focusing on the primary and secondary impairments. Orthoses, for instance, are often used to control the position and movement of the ankle (Novacheck et al., 2009). Neurosurgery, on the other hand, can reduce the patient’s spasticity by means of selective dorsal rhizotomy or intrathecal baclofen, while orthopedic surgery can address bone deformities and muscle contractures (Novacheck and Gage, 2007). Gait analysis has a crucial role in the treatment of gait impairments in CPc, as it allows (1) the identification of the specific deficits of each patient, and (2) the evaluation of the outcome of treatment interventions specifically selected to target those deficits (Schwartz et al., 2004; Novacheck and Gage, 2007).

Interventions in CPc are often assessed via pre–post treatment three-dimensional gait analysis. Until now though, the effect of an alteration in the body weight is usually not taken into consideration when interpreting pre–post data, even though a considerable amount of time can elapse between the two measurements.

However, literature in other populations provides indications that changes in body weight result in alterations in the gait pattern. In children with overweight and obesity for example, it has been reported that additional body mass leads to higher foot loading, with a disproportional impact on the midfoot area (Cousins et al., 2013; Mueller et al., 2016). Both adults and children with obesity have been found to walk with increased peak knee adduction moments (which may result in excessive joint loading) (Gushue et al., 2005; Browning and Kram, 2007). Adults with obesity also have been reported to walk with increased external knee flexion moments, and decreased knee rotation moments compared to non-overweight adults (Harding et al., 2012).

Furthermore, some researchers have indicated changes in EMG activations due to obesity in children and adults as well. Blakemore et al. (2013), showed that during fast walking, overweight children had longer gastrocnemius activity during stance, but shorter gastrocnemius activity during swing (Blakemore et al., 2013). A study by Amiri et al. (2015) indicated that adults with obesity walked with significantly prolonged gastrocnemius and quadriceps EMG activity during the stance phase (Amiri et al., 2015). In adults, walking while carrying load resulted in increased EMG activations of the vastus lateralis and gastrocnemius, as well as increased durations of biceps femoris, gastrocnemius, vastus lateralis, and semimembranosus activity (Ghori and Luckwill, 1985; Simpson et al., 2011).

As such it appears that increased weight has a significant effect on several aspects of the gait pattern, including kinematics, kinetics and EMG.

To the best of our knowledge, the effect of increasing the body weight on the characteristics of gait in CPc has not yet been investigated. To examine this experimentally, one could either gradually increase the weight of CPc over several weeks, or investigate the effect of an immediate increase in weight. One could argue that the gait adaptations to an immediate increase of weight will not necessarily resemble those that happen during the slow increase in body weight as is the case for obesity. In the latter case, the gait pattern can slowly adapt to very small increments in weight. However, body weight can increase more rapidly during daily life as well, i.e., during the growth spurt. During the grow spurt, ambulatory CPc typically show fast increases in body weight, similarly as TDc (Day et al., 2007). In the current study we have opted for the latter, as this is the first step to take to provide further insights into the possible adaptations in CPc to added weight. Thus, the experimental paradigm used in the current study, more closely resembles this phenomenon rather than a slow increase in body weight. As CPc often undergo treatment interventions at an age before or close to the growth spurt, it is of interest to investigate the influence of an immediate increase in weight on the gait parameters in CPc.

The current study examined whether experimentally increasing the body weight influences the spatio-temporal gait characteristics, three-dimensional gait kinematics, kinetics, and EMG activations in CPc. We hypothesize that CPc will experience difficulties in adjusting their gait pattern to the added weights; i.e., they will present with more negative changes in their gait due to the increase of body weight compared to TDc (e.g., decrease walking speed, smaller step lengths and ranges of joint motion). Additionally, we hypothesize that more negative changes in the gait pattern will appear in CPc with relatively weaker lower limb muscles compared to children with relatively strong lower limb muscles as they will experience more difficulties negotiating the increased load imposed on their musculoskeletal system.

## Materials and Methods

### Patients

**FIGURE 1:**
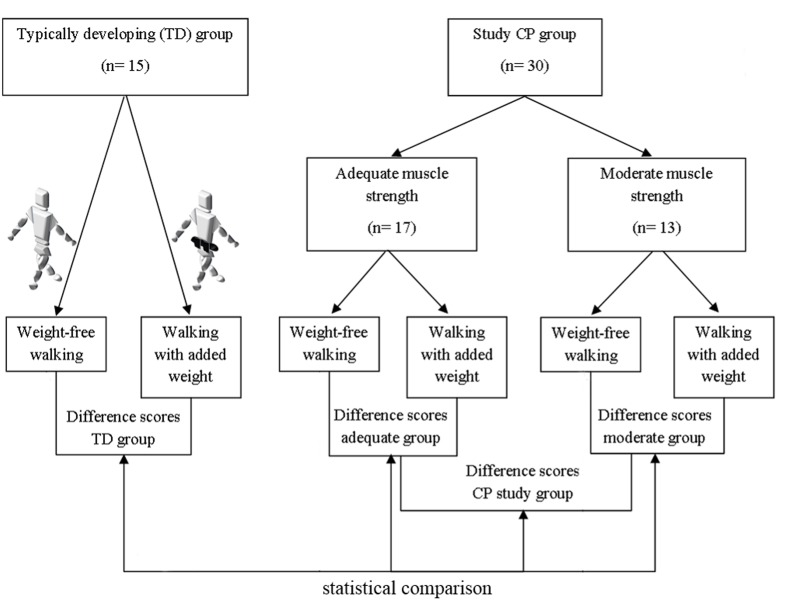
**Schematic overview of the set-up of the study.** The difference scores (walking with added weights vs. weight-free walking) of typically developing (TD) children, all Cerebral Palsy (CP) children from the CP study group, CP children with adequate strength, and CP children with moderate strength are compared in the current study. In the weighted walking task, 10% of the body weight was added evenly around the waist by means of a black belt. The belt was positioned close to the location of S2 to avoid challenging the equilibrium and ensuring full visibility of the markers of the lower limb Plug-in-Gait marker set.

For this study, a group of CPc meeting specific inclusion criteria (*CP group*) was compared to a control group of TDc. The characteristics of both groups are discussed below and summarized in **Table 1**. A schematic overview of the study design can be found in **Figure 1**.

**Table 1:** Participant characteristics.

	CP group		TD group
	Adequate muscle strength group (*n* = 17)	Moderate muscle strength group (*n* = 13)	Typical: typical muscle strength group (*n* = 15)
Average age (SD)	9 y 6 m (±1 y 3 m)	6 y 5 m (±2 y 3 m)	8 y 6 m (±1 y 3 m)
Average weight (SD)	31.8 kg (±10.7 kg)	24.3 kg (±9.3 kg)	29.8 kg (±7.6 kg)
Median (IQR) range of motion (degrees)			
Hip extension	0° (0–0°)	0° (-5–0°)	Nr
Knee extension	0° (0–0°)	0° (0–5°)	Nr
Ankle dorsiflexion	0° (0–10°)	5° (0–10°)	Nr
Selectivity (range 0–2)			
Knee flexors	2 (2–2)	1.5 (1.5–2)	Nr
Knee extensors	2 (2–2)	2 (1.5–2)	Nr
Ankle plantarflexors	1.5 (1.25–2)	1.5 (1–1.5)	Nr
Diagnosis	CP	CP	TD
Unilateral	9	3	Na
Bilateral	8	10	Na
GMFCS			
I	13	4	Na
II	4	9	Na

*SD, standard deviation; y, years; m, months; IQR, interquartile range; range of motion, maximal joint angle achieved passively; Nr, normal; CP, cerebral palsy; TD, typically developing; Na, not applicable; GMFCS, Gross Motor Functional Classification Score.*

For the *CP group*, CPc were recruited at the Laboratory of Clinical Motion Analysis of the University Hospital Leuven (UZ Pellenberg), when they met the following inclusion criteria: (a) predominantly spastic type of CP, (b) age between 3 and 15 years, (c) ability to walk (with or without walking aids), (d) sufficient cooperation to follow verbal instructions, (e) no history of lower limb surgery, (f) no lower limb Botulinum Toxin-A treatment within 6 months prior to the 3DGA, and (g) adequate or moderate lower limb muscle strength. The latter was defined as a MMT-score of at least >2.5 on a 9-point scale (Supplementary Table A) based on the MMT scale developed by Daniels and Worthingham’s scale for manual muscle testing (Daniels and Worthingham, 1986; Cuthbert and Goodheart, 2007). Strength was assessed for the knee flexors, knee extensors, and plantarflexors, as these muscles are assumed to be the main actuators for handling the additional load during the weighted walking (weighted walking task is described below). Based on the median MMT-score for these three muscle groups, the CPc were subdivided into two muscle strength groups: children whose median MMT ≥ 4 were classified into the *adequate* lower limb muscle strength group, while children with a median MMT-score between 2.5 and 4 were classified into the *moderate* lower limb muscle strength group. This cut-off value was selected based on the results of initial pilot tests where it was observed that children with a MMT score > 2.5 were still able to accurately execute the weighted walking task for the full testing procedure. In addition to strength, the selectivity of the three lower limb muscles was assessed using a five-point scale as described by Trost (2009) (Supplementary Table A).

Thirty CPc were enrolled in the *CP group*. Their average age was 8 years 5 months (±2 years 3 months). Twelve children were classified as unilaterally involved while 18 were bilaterally involved. Seventeen children were classified as GMFCS level I and 13 as level II. Seventeen children had *adequate* lower limb muscle strength, while 13 showed *moderate* lower limb muscle strength. Specific lower limb ranges of motion and levels of selective motor control are summarized in **Table 1**.

The TD control group consisted of 15 children with an average age of 8 years 6 months (±1 year 3 months) without a history of orthopedic or neurological pathology. Comparison of the gait adaptations in response to the weighted walking task in the *CP group* to those observed in the TD group enabled the identification of ‘non-typical responses’ (gait adaptations that were not observed in the TD group). Non-typical responses unmask 3D gait parameters that alter due to the addition of weight which are specific to the *CP group*.

To avoid the inclusion of correlated data we studied one of the lower limbs in each participant. Therefore, in CPc with unilateral involvement only the involved lower limb side was considered, while in children with bilateral involvement the most involved side was selected. When both sides were equally involved, the left or right lower limb was randomly selected. For TDc, only the right lower limb was investigated.

All experiments were approved by the local ethical committee and informed, written consent was obtained from the study subjects’ parents.

**Table 2:** Overview of the spatiotemporal, kinematic and kinetic 3DGA parameters that responded significantly different to walking with added weight between typically developing (TD) children and children from the full CP group, CP children with adequate strength (aCP), and CP children with moderate strength (mCP).

	TD		CP		aCP		mCP	
3DGA parameter	Weight-free (±SE)	Weighted (±SE)	Weight-free (±SE)	Weighted (±SE)	Weight-free (±SE)	Weighted (±SE)	Weight-free (±SE)	Weighted (±SE)
Step length (m)	0.54 (0.02)	0.57 (0.03)	0.46 (0.02)	0.44 (0.02)	0.49 (0.02)	0.48 (0.02)	0.42 (0.02)	0.39 (0.03)
Walking velocity (m/s)	1.17 (0.03)	1.28 (0.06)	1.03 (0.04)	0.94 (0.04)	1.09 (0.04)	1.02 (0.05)	0.94 (0.06)	0.83 (0.06)
Stride length (m)	1.07 (0.03)	1.14 (0.05)	0.95 (0.03)	0.89 (0.04)	1.03 (0.04)	0.96 (0.04)	0.84 (0.04)	0.78 (0.05)
Duration of double support (s)	0.18 (0.01)	0.16 (0.01)	0.18 (0.01)	0.22 (0.01)	0.18 (0.02)	0.21 (0.01)	0.17 (0.02)	0.24 (0.03)
Timing of foot-off (% GC)	59.4 (0.5)	58.9 (0.4)	57.9 (0.7)	60.6 (0.7)	58.48 (0.95)	60.35 (0.89)	57.18 (1.07)	60.96 (1.09)
Pelvic rom, cor (°)	7.3 (0.4)	8.0 (0.4)	11.6 (0.7)	9.2 (0.6)	11.57 (1.05)	9.29 (0.89)	11.52 (0.92)	8.97 (0.88)
Max hip fl swing (°)	35.3 (1.1)	39.1 (1.2)	44.6 (1.6)	44.9 (1.6)	44.15 (2.37)	45.41 (2.24)	45.16 (1.90)	44.28 (2.16)
Hip rom, sag (°)	45.3 (1.1)	50.1 (1.3)	46.9 (1.5)	48.1 (1.8)	47.22 (2.50)	47.26 (2.58)	46.54 (1.37)	49.17 (2.52)
Hip rom, cor (°)	12.6 (0.7)	14.1 (0.6)	14.4 (0.7)	13.6 (0.9)	14.68 (1.12)	13.22 (1.15)	13.98 (0.83)	14.09 (1.34)
Hip rom, trans (°)	21.3 (1.0)	25.6 (1.6)	21.7 (1.1)	20.1 (1.0)	23.47 (1.10)	21.63 (0.85)	19.40 (1.91)	18.03 (1.99)
Max hip fl vel in swing (°/s)	220.5 (6.6)	251.5 (10.0)	223.6 (9.1)	229.8 (11.2)	226.1 (14.2)	229.3 (16.8)	220.5 (10.5)	230.5 (14.6)
Max plfl vel at toe-off (°/s)	161.9 (12.8)	200.9 (10.2)	127.0 (7.8)	121.2 (9.8)	128.4 (10.4)	116.0 (10.5)	125.1 (12.2)	128.1 (18.3)
Max hip ext moment in stance (Nm)	0.50 (0.08)	0.98 (0.07)	0.99 (0.07)	0.80 (0.07)	1.11 (0.09)	0.90 (0.09)	0.79 (0.09)	0.62 (0.10)
Max hip power gen in stance (W)	0.49 (0.09)	0.76 (0.07)	1.36 (0.16)	1.07 (0.14)	1.42 (0.22)	1.18 (0.20)	1.25 (0.21)	0.88 (0.15)
Max hip power abs in stance (W)	-0.69 (0.05)	-0.97 (0.08)	-0.97 (0.09)	-0.94 (0.09)	-0.99 (0.11)	-1.01 (0.12)	-0.92 (0.16)	-0.82 (0.14)
Max hip power gen at toe-off (W)	1.30 (0.09)	1.65 (0.16)	1.15 (0.11)	1.08 (0.14)	1.32 (0.14)	1.33 (0.19)	0.85 (0.16)	0.63 (0.10)
Max knee power gen in stance (W)	0.54 (0.06)	0.99 (0.13)	0.89 (0.14)	0.89 (0.16)	1.00 (0.20)	1.01 (0.23)	0.69 (0.13)	0.67 (0.11)
Max ankle power abs at loading response (W)	-0.63 (0.08)	-0.73 (0.08)	-0.96 (0.09)	-0.82 (0.08)	-0.95 (0.12)	-0.83 (0.10)	-0.97 (0.15)	-0.81 (0.14)

*The values of these parameters for both baseline (weight-free) walking trials and weighted walking trials are presented.*

*TD, typically developing; CP, cerebral palsy; 3DGA, 3D gait analysis; SE, standard error; rom, range of motion; cor, coronal plane; sag, sagittal plane; trans, transversal plane; max, maximal; fl, flexion; plfl, plantarflexion; ext, extension; vel, velocity; gen, generation; abs, absorption.*

**Table 3:** Statistically significant 3DGA parameters and their level of significance per statistical comparison.

	Mann–Whitney *U*-values, *P*-values and Effect Sizes of comparisons based on difference scores (between baseline walking and weighted walking)
	
3DGA parameter	TD vs. CP	TD vs. aCP	TD vs. mCP	aCP vs. mCP
Step length (m)	89 (0.03)/-0.45	ns	ns	ns
Walking velocity (m/s)	81 (0.02)/-0.49	ns	ns	ns
Stride length (m)	86 (0.02)/-0.47	ns	ns	ns
Duration of double support (s)	74 (0.02)/-0.49	ns	ns	ns
Timing of foot-off (% GC)	56 (<0.01)/-0.59	40 (<0.01)/-0.57	16 (<0.01)/-0.68	ns
Pelvic rom, cor (°)	55 (<0.01)/-0.61	23 (<0.001)/-0.70	32 (<0.01)/-0.57	ns
Max hip fl swing (°)	64 (<0.01)/-0.58	53 (<0.01)/-0.50	11 (0.001)/-0.75	ns
Hip rom, sag (°)	113 (0.03)/-0.40	ns	ns	ns
Hip rom, cor (°)	108 (0.03)/-0.42	ns	ns	ns
Hip rom, trans (°)	92 (0.02)/-0.48	ns	ns	ns
Max hip fl vel in swing (°/s)	108 (0.03)/-0.42	ns	ns	ns
Max plfl vel at toe-off (°/s)	91 (0.02)/-0.48	ns	ns	ns
Max hip ext moment in stance (Nm)	28 (<0.001)/-0.70	15 (<0.001)/-0.75	13 (<0.01)/-0.66	ns
Max hip power gen in stance (W)	82 (0.02)/-0.47	ns	ns	ns
Max hip power abs in stance (W)	89 (0.03)/-0.44	ns	ns	ns
Max hip power gen at toe-off (W)	88 (0.03)/-0.44	ns	ns	ns
Max knee power gen in stance (W)	89 (0.03)/-0.40	ns	ns	ns
Max ankle power abs at loading response (W)	96 (0.04)/-0.42	ns	ns	ns
Mean EMG ampl gastroc increase/decrease (%)	108 (0.03)/-0.42	ns	ns	ns

*Comparisons were based on the difference scores between baseline walking and walking with added weights (for the data, see **Table 2**).*

*Data is presented as follows; *U*-value (*p*-value)/Effect size. 3DGA, 3D gait analysis; TD, typically developing; CP, cerebral palsy study group; aCP, adequate strength CP group; mCP, moderate strength CP group; ns, not significant; na, not applicable; rom, range of motion; cor, coronal plane; sag, sagittal plane; trans, transversal plane; max, maximal; fl, flexion; plfl, plantarflexion; ext, extension; vel, velocity; gen, generation; abs, absorption; ampl, amplitude; gastroc, gastrocnemius muscle.*

### 3D Gait Analysis and Weighted Walking Task

Gait was evaluated through 3D gait analysis (3DGA). Prior to the 3DGA, lower limb dimensions, body height, and weight were measured to enable an estimation of joint center locations and segmental inertia parameters. Patients walked on a 10-meter walkway at a self-selected speed. Spatiotemporal and kinematic measurements were collected using an eight-camera VICON system (612 data capturing system measuring at 100 Hz; VICON, Oxford Metrics, Oxford, UK), with the lower limb Plug-In-Gait marker set (Kadaba et al., 1990). Two force plates (Advanced Mechanical Technology Inc., Watertown, MA, USA) were embedded in the walkway for force registration enabling calculation of kinetics. Surface EMG data were collected from the medial hamstrings, rectus femoris, and lateral gastrocnemius muscles using a 16 channel EMG system (K-lab, Biometrics, The Netherlands; or Zerowire, Aurion, Italy), synchronized with the motion capture system. EMG data was collected with a sampling rate of 1500 Hz and filtered with a zero-phase 6th order butterworth filter with a passband ranging from 20 to 500 Hz. Nexus software (Oxford Metrics, Oxford, UK) was used to define the gait cycles, to calculate the spatiotemporal parameters, and to estimate the joint angles and internal moments and powers normalized to body mass. Seventy-one discrete parameters were extracted from the continuous kinematic and kinetic waveforms based on a study of the literature (Inman et al., 1981; Winter, 1987; Perry, 1992; Schutte et al., 2000; Goldberg et al., 2006; Wolf et al., 2006) and the routine gait analysis protocol used for children with CP at the Clinical Movement Analysis Laboratory of the University Hospital Leuven (Pellenberg).

All children walked barefoot (baseline/weight-free walking). In addition, all children executed the weighted walking task consisting of barefoot walking with 10% of the body weight added around the waist. This percentage of body weight was selected based on initial pilot tests (*n* = 9). In these tests, it was observed that adding 10% of the body weight resulted in an increased load on the lower limbs without overloading, assuring the ability to walk across the gait lab for the full test procedure. The results from these pilot tests indicated that adding more than 10% of the body weight limited some patients to perform the walking trials. Hence, 10% of the body weight was chosen. The weight was applied evenly around the waist by means of a belt. The belt was positioned close to the level of S2 (approximate location of the center of gravity) to avoid challenging the equilibrium of the children, and above the marker of the pelvis to ensure full visibility of the lower limb Plug-In-Gait marker set. For both conditions, the children walked at a self-selected speed until three trials with full kinematics, kinetics and EMG were recorded. Internal moments and powers for the weighted walking trials were normalized to the child’s body weight plus the additional weight.

### Statistical Analysis

For this study, a subset of 71 gait parameters was selected from the output of the 3DGA for both walking conditions (baseline and weighted walking). Spatiotemporal parameters, discrete values of joint angles, moments and powers at specific points in the gait cycle, and the mean EMG frequency over one full gait cycle were automatically extracted from the gait waveforms by means of custom-made software implemented in MATLAB (Mathworks). All parameters were averaged per walking condition over the three registered walking trials. Additionally, the percentage increase/decrease in mean EMG amplitude (raw signal) in response to the added weight was calculated. All EMG parameters were investigated for the medial hamstrings, rectus femoris, and lateral gastrocnemius muscles.

The difference scores between baseline walking and weighted walking were calculated for all the 3DGA parameters of the *CP group* and the TD group. These scores illustrate how the children responded to an increase in body weight. A Kruskal–Wallis test with *post hoc* Mann–Whitney *U* tests [with False Discovery Rate (FDR) correction for multiple testing] compared the responses of the TD children to the responses of (1) all CPc in the *CP group*, (2) CPc with adequate strength, and (3) CPc with moderate strength. A non-parametric method was applied as not all outcome parameters were characterized by a normal or Gaussian distribution. All statistical procedures were performed with the SAS system (SAS Institute Inc, SAS Campus, Dr. Cary, NC 27513). Level of significance was set at *P* < 0.05.

## Results

When children walked with added weight, changes in the gait pattern were observed for both TDc and CPc. However, for several 3DGA parameters, CPc responded significantly different to the added weight when compared to TDc. **Tables 2** and **3** provide detailed results.

**FIGURE 2:**
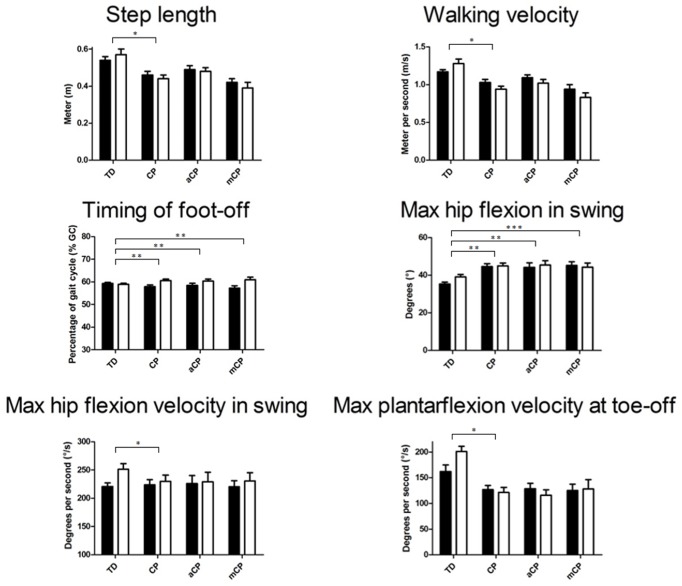
**Mean (+SE) 3D gait analysis parameters that significantly differed in response to walking with added weight between TD children and children with CP and/or between TD, CP children with adequate strength (aCP) and CP children with moderate strength (mCP).** Black bars represent the weight-free trials, white bars represent the weighted walking trials. See **Tables 2** and **3** for specific significance values. m, meter; % GC, percentage of gait cycle; m/s, meter per second; Gas, gastrocnemius muscle; ampl, amplitude. ^∗^*p* ≤ 0.05, ^∗∗^*p* ≤ 0.01, ^∗∗∗^*p* ≤ 0.001.

Overall, the TDc appeared to increase their walking velocity, step- and stride length, and decreased their duration of double support while the timing of foot-off decreased slightly, while the opposite pattern was found in CPc (**Figure 2**; **Table 2**).

Increased ranges of motion at the pelvis (coronal plane) and hip (all planes), as well as higher joint angular velocities at the hip and ankle appeared for TDc, while CPc did not seem to change or even decreased the respective ranges of motion and joint angular velocities in response to walking with added weight (**Figure 2**). Furthermore, several moments and powers at the hip, knee, and ankle appeared to increase in TDc, while CPc mainly seemed to decrease their moments and powers during weighted walking (**Tables 2** and **3**).

**FIGURE 3:**
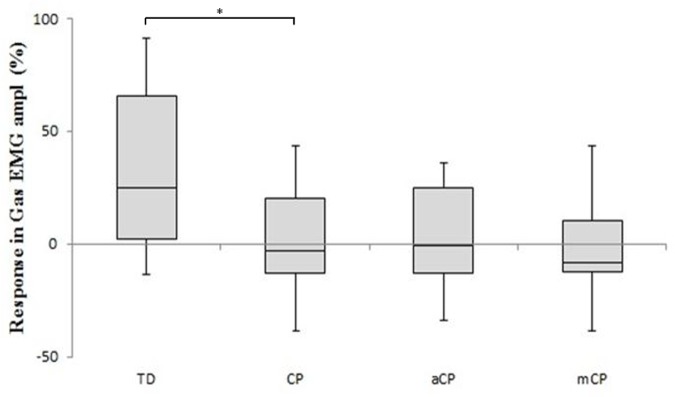
**The EMG amplitude difference scores [mean (+SE)] of the gastrocnemius muscle for the TD children and the children with CP in response to walking with added weights.** Comparisons are made to the full CP study group and separately to the two muscle strength CP sub-groups: aCP, adequate lower limb strength; mCP, moderate lower limb strength. Difference scores are expressed as percentage increase/decrease. ^∗^*p* ≤ 0.05.

Finally, a significantly different response in EMG amplitude could be observed in the gastrocnemius. In general, TDc increased their gastrocnemius EMG amplitude when they walked with added weight, while CPc slightly decreased their gastrocnemius EMG amplitude (**Figure 3**).

When the TD group was compared to the subgroups representing the adequately and moderately strong CP children separately, the response of four 3DGA parameters (timing of foot-off, pelvic range of motion in the coronal plane, maximal hip flexion in swing and maximal hip extension moment in stance) was found to differ significantly confirming the above described results (**Tables 2** and **3**). No differences in gait parameters due to the increased weight were found between the two CP groups (the adequately and moderately strong CPc).

## Discussion

Walking with extra weight applied around the waist resulted in multiple changes in the gait pattern of both TDc and CPc. For several 3DGA parameters, these changes were significantly different between the TDc and the *CP group*.

The current results are of clinical importance as, due to the increase in prevalence of overweight and obesity in CPc over the last decades (Rogozinski et al., 2007; Park et al., 2011), the gait pattern in CPc to date can be negatively influenced. Consequently, when interpreting 3D gait analysis data, weight should also be considered as a potential factor influencing the gait parameters.

While TDc seemed to be able to successfully handle the extra weight, CPc experienced difficulties to walk with the increased demand. TDc walked with larger ranges of joint motion, and higher moments and powers in response to the added weight (**Table 2**; **Figure 2**). In fact, it appeared that TDc even overcompensated for the extra weight as their resulting gait was faster and more dynamic (reflected in increased kinetics). In contrast, CPc walked slower, with smaller step lengths and ranges of joint motion, and decreased moments and powers. Furthermore, while in TDc the EMG amplitude of the gastrocnemius muscle increased as a result of the added weight, in CPc the EMG amplitude slightly decreased (**Figure 3**). To increase its force output, a muscle can apply two strategies (or a combination of both). Either more motor units are recruited, or the activity of already recruited motor units is synchronized. The first strategy will increase both the EMG frequency and amplitude, while the latter decreases the EMG frequency whilst increasing the amplitude (Lauer et al., 2008). As the EMG amplitude slightly decreased in CPc, it appears that in CP the muscles failed to successfully increase their force output during weighted walking. This finding is supported by the conclusions in the study of Dallmeijer et al. (2011) who found that the lower limb muscles of CPc are characterized by a small muscle reserve during walking (Dallmeijer et al., 2011).

When the CP group was further subdivided into children with adequate and moderate lower limb muscle strength, no significant differences in 3DGA parameters were found due to the added weight in the two subgroups. As such, it appears that the difference in muscle strength between the two CP strength subgroups did not lead to a significantly different response to the added weight. Nevertheless, both CP subgroups presented with difficulties to walk as a result of an increase in weight.

Previous research already provided an indication that weight could have a negative impact on the gait pattern in CPc. Specifically, the sudden increase in body weight during the pubertal growth spurt has been previously related to a more rapid development of the crouch gait pattern in children with diplegic CP (Gage, 2004). This rapid development was suggested to be related to increased body mass with an unfavorable mass-to-strength ratio; i.e., weak muscles can no longer support the toe walking pattern due to the sudden increase in weight (Kedem and Scher, 2016).

The finding that an increase in body weight has significant negative consequences on several gait parameters in CPc that are frequently used in clinical gait analysis (and the overall gait pattern) is of high clinical importance. Given the fact that interventions in CPc are often evaluated via pre- and post 3D-gait analyses, clinical gait analysts should take into account the increase in weight of the patient, definitely if the assessments take place during or close to the growth spurt. If not, unfavorable (or less favorable than expected) gait outcomes could be related to the treatment rather than to the increase in body weight.

These findings underscore the importance of initiatives such as that of the European Union and the World Health Organization to attempt to cease the rise in overweight and obesity in children (European Union, 2014; World Health Organization, 2016a). These initiatives are of vital importance as they can halt the related health problems associated to overweight, but also stop the negative influence of overweight on the gait pattern in CPc.

When interpreting the results of the current study, one should consider some possible limitations.

It could be argued that similar changes in spatiotemporal parameters of gait have been previously reported as specific markers of balance problems (Liao et al., 1997). However, the weighted walking task of the current study was specifically designed to increase the body weight without challenging the equilibrium by adding the weight evenly around the waist and close to the center of gravity. Nevertheless, it cannot be ruled out that there was no effect on balance. Further research could focus on further elucidating the individual role of balance on (spatiotemporal) gait parameters during weighted walking.

The decrease in EMG amplitude in CPc could also be partly related to muscle fatigue. It has been described in healthy adults that muscle fatigue influences the EMG signal and, e.g., reduces its frequency (Rogers and MacIsaac, 2011). Fatigue could have played a part in the setup of the current study, as the muscle endurance during submaximal tasks in CPc is reduced compared to TDc (Eken et al., 2014). Hence, the combination of the different walking trials with and without weights, and the reduced capacity of CPc to endure activities could have induced muscle fatigue. Combined with the fact that CPc have been shown to have a small muscle reserve during walking (Dallmeijer et al., 2011), this means that the EMG amplitude could not be increased in CPc. Further research examining the mean EMG frequencies and amplitudes of lower limb muscles during gait for several consecutive steps of a large group of CP children might further elucidate this problem.

In the current study, the participants’ response was investigated when the weight of the body was experimentally/artificially increased. This allowed us to investigate fundamental changes in the gait pattern in TDc and CPc as a response to a sudden increase in body weight. It could, however, be argued that an increase in body weight in daily life (as happens with overweight and obesity) occurs over a long period of time in which the gait pattern can slowly adapt. Even though the participants in the current study received some practice trials with the weights (until they indicated to feel comfortable with the weights), it is possible that a longer period of adaption could have an impact on the gait pattern changes. Nevertheless, results from previous studies in people with overweight and obesity support our findings (e.g., increase in EMG activations, and knee power generation in TDc). Furthermore, our experimental setup could be specifically interesting in the case of a more rapid increase in body weight which occurs during the growth spurt (Day et al., 2007). In this case, however, one should consider that the current setup does not take into account the combination of the lengthening of the body combined with the increase in body weight. Hence, the weight increase during the growth spurt is spread over the body rather than focused on the trunk.

## Conclusion

The results of the current paper indicate that a multitude of gait parameters (including spatiotemporal measures, kinematics, kinetics, and EMG) are significantly influenced by adding 10% body weight during walking in TDc and CPc. Furthermore, many of these gait parameters differed significantly between TDc and CPc in response to the added weight. Given the rapid increase in body weight during the growth spurt in CPc, specialists in clinical gait analysis should take into account the negative effect of the increased body weight during pre–post measurements to avoid misinterpretation of the treatment results. Overweight and obesity in CPc should be counteracted or prevented as the increased weight has a detrimental effect on their gait pattern (and other health issues).

## Author Contributions

LVG and KD conceived and designed the experiment. GM, PDC, EO, and LVG performed patient recruitment. PM and LVG tested the participants. PM, LVG, and LB-O performed statistical analyses and interpreted the data. HW, EA, and HB created the software for the data extraction of the three dimensional gait analyses. LVG, MG, HW, and EA performed data quality checks. PM, LVG, and KD wrote the paper. MG performed the lay-out and edited the paper. The writing process and data analysis were supervised by KD.

## Conflict of Interest Statement

The authors declare that the research was conducted in the absence of any commercial or financial relationships that could be construed as a potential conflict of interest.
