# Supplementary material for: Children with Spastic Cerebral Palsy Experience Difficulties Adjusting Their Gait Pattern to Weight Added to the Waist, While Typically Developing Children Do Not
Source: Front Hum Neurosci. 2016 Dec 23;10:657. doi: 10.3389/fnhum.2016.00657 (PMC5226450; doi:10.3389/fnhum.2016.00657)
Supplement: Supplementary file 1 [file Table_1.DOCX]

Table A

| Muscle strength evaluation (based on Cuthbert & Goodheart, 2007; Daniel & Worthingham, 1986) | | |
| --- | --- | --- |
| Score |  | Criteria to be met |
| 0 |  | Contraction cannot be palpated |
| 1 |  | Evidence of slight contraction of the muscle but joint motion is not visible |
| 1.5 |  | Initiates motion if gravity is eliminated |
| 2 |  | Complete range of motion in gravity eliminated plane (available ROM, ROM can be slightly decreased because of cocontraction) |
| 2.5 |  | Incomplete ROM against gravity (almost perfect motion against gravity, incomplete range, motion with little help) |
| 3 |  | Perfect motion against gravity (almost full available ROM, ROM can be slightly decreased because of cocontraction) |
| 3.5 |  | Motion against gravity with minimal resistance (almost full available ROM, ROM can be slightly decreased because of cocontraction) |
| 4 |  | Motion against gravity with some (moderate) resistance (full available ROM) |
| 5 |  | Motion against gravity with maximal resistance (full available ROM) |
| Muscle selectivity evaluation (Gage et al., 2009) | | |
| Score |  | Criteria to be met |
| 0 |  | No selective control, no (or minimal) contraction of the demanded muscles |
| 0.5 |  | Small contraction but almost no motion, and/or a lot of cocontraction |
| 1 |  | Mild selective control, not all muscles working in a correct way, no smooth motion, with cocontracion (not always), limited ROM |
| 1.5 |  | Good contraction, with correct muscles, but slightly limited range because of cocontraction or no perfect smooth motion |
| 2 |  | Perfect control, perfect contraction with correct muscles |

ROM= range of motion
